# Comparative metabolomic profiling using untargeted UHPLC–HRMS and GC–MS reveals thermal-induced chemical changes in dried turmeric

**DOI:** 10.1016/j.fochx.2025.103104

**Published:** 2025-10-01

**Authors:** Choong-In Yun, Ga-Yeong Lee, Young-Jun Kim, JaeHwan Lee

**Affiliations:** a Department of Food Science and Biotechnology, Sungkyunkwan University, Suwon 16419, Republic of Korea; b Department of Food Science and Biotechnology, Seoul National University of Science and Technology, Seoul 01811, Republic of Korea; c Research Institute of Food and Biotechnology, Seoul National University of Science and Technology, Seoul 01811, Republic of Korea

**Keywords:** *Curcuma longa*, Ulgeum, Untargeted metabolomics, Thermal processing, Bioactive compounds, Sesquiterpenes

## Abstract

This study examined the effects of heat treatment on the bioactive compound profiles of of dried *Curcuma longa* cultivated in different regions. Korean-cultivated *C. longa* (ulgeum) is typically grown as a tuberous root, whereas Indian-cultivated *C. longa* (turmeric) develops as a rhizome, reflecting differences in cultivation practices and environmental conditions. Through untargeted metabolite profiling using UHPLC–HRMS and GC–MS, the research identified major changes in the metabolome after thermal processing, including the formation of bioactive compounds associated with the degradation of curcuminoids and turmerones. Multivariate statistical analyses including PCA and ANOVA, and heatmap cluster analysis demonstrated distinct metabolic responses between the *C. longa* varieties. Notable heat-induced metabolites, including vanillin, dehydrozingerone, α-methylcinnamic acid, and β-elemenone, were identified. These findings indicate that heat treatment not only impacts the stability of bioactive compounds but also improves the potential of heat-treated *C. longa* in food and functional food applications by generating highly bioactive metabolites.

## Highlights

• Heat treatment altered the metabolite profiles of Turmeric (*C. longa*).
• Major thermal degradation products were identified using UHPLC–HRMS and GC–MS.
• Multivariate analyses showed metabolic differences among the two varieties.
• Heat-induced metabolites, including vanillin and β-elemenone, were identified.
• An in silico study was conducted to evaluate the theoretical absorption percentage.

## Graphical abstract

**Figure:**
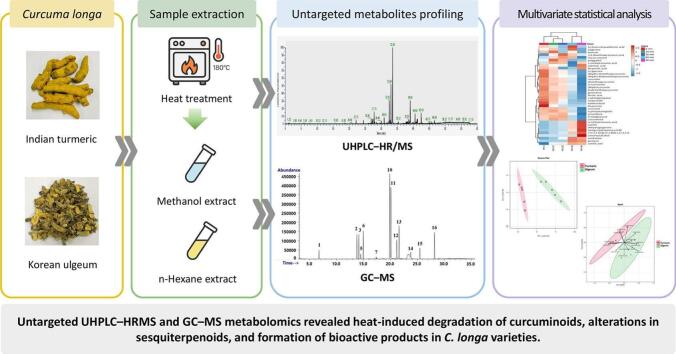

## 1 Introduction

Turmeric (*Curcuma* spp.), a spice derived from the ginger family (Zingiberaceae), is widely recognized for its medicinal properties, owing to its bioactive compounds, particularly curcuminoids (Kim, Lee, & Shin, 2013). Its medicinal properties include antioxidant, antimicrobial, antimalarial, anti-inflammatory, antitumor, and anti-aging effects (Avanço et al., 2017; El-Saadony et al., 2023; Jikah & Edo, 2024; Sharifi-Rad et al., 2020). In addition to curcuminoids, turmeric contains various bioactive compounds, including phenolic acids, flavonoids, terpenoids, phenylpropanoids, and sesquiterpenes, all of which contribute to its health-promoting properties (Jikah & Edo, 2024; Núñez, Vidal-Casanella, Sentellas, Saurina, & Núñez, 2020). Furthermore, the presence of these compounds enhances the therapeutic potential of turmeric as a valuable ingredient in traditional and contemporary food applications (Ballester et al., 2023; Jyotirmayee et al., 2024).

Korean-cultivated *Curcuma longa* (ulgeum) is typically cultivated as a tuberous root, whereas Indian-cultivated *Curcuma longa* (turmeric) develops more extensively as a rhizome. This distinction arises from differences in cultivation practices and environmental conditions between the two regions. This distinction can be attributed to differences in cultivation practices and environmental conditions between the two regions. Korea's shorter growing season and cooler climate tend to promote tuberous root development in *C. longa*, whereas India's longer growing period and warmer climate are more favorable for extensive rhizome formation (Lee et al., 2020). In practical use, Korean ulgeum is generally distributed as dried slices or ground powder and is mainly consumed as herbal teas and beverages (Lee et al., 2020). In contrast, Indian turmeric is commonly marketed as finger-like dried rhizomes or ground powder and is widely used as a culinary spice and as an ingredient in functional food products (Ballester et al., 2023; Jikah & Edo, 2014). Several studies have reported that Korean ulgeum exhibits a distinct phytochemical profile compared to Indian turmeric, including lower curcumin concentrations and higher levels of essential oils such as *ar*-turmerone (Choi, 2009; Hwang et al., 2016). These findings are consistent with recent analyses of Korean ulgeum, which revealed elevated contents of sesquiterpenoids like α-zingiberene and β-sesquiphellandrene, further distinguishing it chemically from Indian varieties (Hwang et al., 2016; Umar, Parumasivam, Aminu, & Toh, 2020; Yun, Yang, Lee, & Kim, 2024).

Bioactive compounds in *Curcuma* spp., including curcuminoids, phenolic acids, flavonoids, terpenoids, phenylpropanoids, sesquiterpenes, and essential oils, have been characterized and identified using various analytical methodologies (Elhawary, Moussa, & Singab, 2024; Feng, 2022; Jyotirmayee et al., 2024; Karpuz Ağören & Akkol, 2024; Kotha & Luthria, 2019; Núñez et al., 2020). Gas chromatography–mass spectrometry (GC–MS) has been shown effective for profiling volatile components in *Curcuma* spp., such as essential oils and terpenoids, providing detailed chemical fingerprints for sample authentication and differentiation (Núñez et al., 2020). Liquid chromatography–tandem mass spectrometry (LC–MS/MS) has been widely used for the targeted quantification of bioactive compounds such as curcuminoids, phenolic acids, and flavonoids, owing to its high sensitivity and specificity (Jyotirmayee et al., 2024). However, despite its effectiveness for targeted profiling, its limited mass accuracy and resolving power restrict its ability to comprehensively characterize complex mixtures. To overcome these constraints, high-resolution mass spectrometry (HRMS) platforms, such as Time-of-Flight (TOF) and Orbitrap analyzers, have been increasingly utilized. These advanced instruments provide exceptional mass accuracy and resolving power, facilitating the accurate determination of molecular masses and the resolution of isobaric compounds. In the context of metabolomic profiling of complex plant matrices, HRMS enables high-confidence identification of metabolites, thereby surpassing the analytical capabilities of conventional LC-MS/MS systems (Feng, 2022). HRMS complements these methods by delivering precise mass measurements of curcuminoids and related compounds, even at low concentrations, ensuring accurate identification and quantification (Kotha & Luthria, 2019).

Table S1 summarizes previous studies that have utilized HRMS for the characterization, identification, metabolite profiling, and metabolomics-based discrimination of *Curcuma* spp. (Awin et al., 2019; Klau et al., 2023; Liu et al., 2016; Núñez et al., 2020; Salem, El-Shiekh, Fernie, Alseekh, & Zayed, 2022; Septaningsih, Darusman, Afendi, & Heryanto, 2018). Despite these advances, relatively little attention has been paid to how thermal processing alters the chemical composition and metabolic profile of *C. longa*, even though heat treatment is a typical step in cooking and industrial processing (Zagórska, Kukula-Koch, Czop, Iłowiecka, & Koch, 2023; Zhu, Sanidad, Sukamtoh, & Zhang, 2017). Heat processing substantially influences the stability and bioactivity of *C. longa* metabolites, as curcuminoids are highly thermolabile due to the instability of the central β-diketone moiety and undergo hydrolytic and oxidative degradation into products such as vanillin, ferulic acid, and dehydrozingerone, which have also been reported to retain biological activities including antioxidant and anti-inflammatory effects (Esatbeyoglu, Ulbrich, Rehberg, Rohn, & Rimbach, 2015; Slavova-Kazakova, Koleva, Kancheva, & Delogu, 2018). Sesquiterpenes such as ar-turmerone also gradually degrade through oxidation, leading to reduced abundance and altered functional properties (Dosoky, Satyal, & Setzer, 2019; Li et al., 2011). However, recent metabolomic studies investigating the chemical changes in *C. longa* induced by heat treatment remain insufficient. Numerous studies have focused on the quantitative analysis of phytochemicals in *C. longa*, particularly curcuminoids. In contrast, its rich essential oil fraction, composed largely of sesquiterpenes such as *ar*-turmerone, has been primarily investigated through qualitative analyses and compositional profiling. However, previous studies combining ultra-high-performance liquid chromatography (UHPLC)–HRMS and GC–MS to comprehensively profile two *C. longa* varieties with distinct bioactive compound characteristics are still lacking.

Therefore, this study is structured to investigate the effects of heat treatment on the bioactive compound profiles of Korean ulgeum and Indian turmeric, two varieties expected to exhibit distinct phytochemical characteristics. First, untargeted metabolite profiling of *C. longa* was performed using UHPLC–HRMS, and complemented by GC–MS analysis to profile lipophilic compounds such as sesquiterpenes and essential oils. Recognizing the low quantitative reproducibility of GC–MS, an internal standard (IS) was incorporated to enhance analytical accuracy and reliability. The resulting peak signal profiles served as the sources for multivariate statistical analysis. Differences in peak areas were examined with respect to various heat treatment durations by principal component analysis (PCA) and heatmap cluster analysis. This approach enabled discrimination between the two *C. longa* varieties and allowed the identification of compounds that either decreased or increased during heat treatments, as revealed by the PCA loading plot and analysis of variance (ANOVA).

This study is expected to provide valuable insights into the impact of heat treatment on the stability and transformation of bioactive compounds in *C. longa*, which is commonly consumed in heat-processed forms. By understanding how heat processing influences the safety and bioactivity of these compounds, this research aims to identify potential degradation products that could exhibit bioactive properties. Additionally, the evaluation of newly identified compounds for their theoretical absorption percentage and Caco-2 permeability is anticipated to highlight their contribution to the overall bioactivity of *C. longa*. These findings are expected to offer a comprehensive understanding of *C. longa*'s functional properties post-heat treatment, supporting its continued use as a nutraceutical ingredient and providing a scientific basis for its applications in heat-processed food products.

## 2 Materials and methods

### 2.1 Chemicals and materials

Ethyl octanoate and formic acid were purchased from Sigma-Aldrich (St. Louis, MO, USA). High-performance liquid chromatography (HPLC)-grade water, methanol, *n*-hexane, and acetonitrile were obtained from J.T. Baker (Phillipsburg, NJ, USA). Reference standards were used for metabolite annotation for seven compounds: bisdemethoxycurcumin, demethoxycurcumin, and curcumin, purchased from Toronto Research Chemicals (Toronto, Canada), while *ar*-turmerone and 2-methoxy-4-vinylphenol, vanillin, vanillylidenacetone (dehydrozingerone) obtained from Sigma-Aldrich (St. Louis, MO, USA). For this study, dried *C. longa* samples were procured from the Korean herbal medicine market in Seoul, South Korea, in 2023. The samples were purchased from verified suppliers, who provided certificates of analysis confirming their quality and safety, including morphological identification, heavy metal content, pesticide residues, and other relevant quality control parameters. The botanical identity of Korean ulgeum is not clearly defined, as previous studies have inconsistently described it as either *C. longa* or *C. aromatica*. To clarify the species identity, genomic DNA was extracted from powdered rhizome tissue using a CTAB-based method with reagents purchased from Sigma-Aldrich (St. Louis, MO, USA), following the protocol described by Chen et al. (2023). The ITS2 and chloroplast trnK–matK regions were amplified using primer sets (ITS2ZF/ITS8ZR and trnK3914F/CT828R) designed by Chen et al. (2023) and synthesized by Takara Korea (Seoul, South Korea). PCR reactions were carried out using a commercial PCR Master Mix (Takara Bio, Shiga, Japan). The resulting amplicons were purified and subjected to Sanger sequencing by Macrogen (Seoul, South Korea). Subsequent BLASTn analysis against the NCBI GenBank database (https://blast.ncbi.nlm.nih.gov/Blast.cgi) revealed high sequence similarity (99.9–100 %) to *C. longa* accessions (e.g., MW838984.1 and AB047738.1), thereby confirming the species identity of both Korean ulgeum and Indian turmeric as *C. longa*. Since *C. longa* is commonly marketed as dried rhizomes, this study simulated additional thermal processing to reflect typical post-harvest handling conditions. Dried *C. longa* was used as the control matrix to represent the form commonly encountered by consumers and manufacturers, and to investigate heat-induced metabolic changes relevant to cooking or industrial applications. The samples included the dried rhizome of *C. longa* (commonly known as turmeric, most frequently exported from India into South Korea) and the dried tuberous root of *C. longa* (ulgeum in Korean, a native species cultivated in South Korea) to identify the chemical compounds according to heat treatment duration.

### 2.2 Heat treatment condition and extraction

Dried *C. longa* (0.5 g each) were accurately weighed and placed into 50 mL amber volumetric flasks. The samples were subjected to heat treatment in a dry oven at 180 °C for 0, 10, 30, 60, and 90 min with the 0-min sample representing the un-heat-treated control. After heating, the samples were allowed to cool to room temperature. The heating temperature and time were determined based on literature reporting that most of the curcumin, the primary component of *C. longa*, degrades at 180 °C within 70 min (Esatbeyoglu et al., 2015). Accordingly, this study set the heat treatment to 90 min or less. For UHPLC–HRMS analysis, the extraction was performed using 50 mL of methanol with ultrasonication (50 kHz, 500 W). For GC–MS analysis, the extraction was conducted using 50 mL of *n*-hexane with ultrasonication. The extraction method used in this study was adapted from a previous study (Xu et al., 2020). After extraction, the supernatants were filtered through 0.22 μm regenerated cellulose (RC) membrane filters (Sartorius, Göttingen, Germany) to eliminate particulates. When immediate analysis was not possible, the filtered extracts were stored in amber vials at −80 °C until analysis. To improve the low reproducibility of GC–MS peak areas, ethyl octanoate was added as an IS to enhance analytical accuracy. A solution of ethyl octanoate was prepared at a concentration of 100 mg/L in *n*-hexane. For each analysis, 0.05 mL of the IS solution was added to 0.95 mL of the sample extract, and the mixture was thoroughly homogenized before injection into the GC–MS system.

### 2.3 Analytical instrument

#### 2.3.1 UHPLC–HRMS conditions

The UHPLC–HRMS conditions for the identification of chemical compounds were optimized by modifying the protocol described in a previous study (Kim, Park, Yun, & Kim, 2024). The chromatographic separation was carried out using a Thermo Scientific™ Vanquish Flex UHPLC System (Thermo Fisher Scientific, San Jose, CA, USA) equipped with a Waters CORTECS T3 column (2.1 × 150 mm, 1.6 μm; Waters Corp., Milford, MA, USA) with the column temperature maintained at 45 °C. The mobile phase consisted of solvent A (0.1 % formic acid in deionized water) and solvent B (0.1 % formic acid in acetonitrile) at a constant flow rate of 0.25 mL/min. A gradient elution program was set up as follows: 3 % solvent B was maintained from 0 to 0.1 min, gradually increased to 15 % solvent B over 15 min, followed by a linear increase to 100 % solvent B between 50 and 55 min. At 55.1 min, the gradient returned to 3 % solvent B and was maintained until 60 min for column re-equilibration. Both MS and MS/MS analysis were performed using a Thermo Fisher Scientific Q-Exactive™ Orbitrap mass spectrometer (Thermo Scientific, Horsham, Loughborough, and Inchinnan, UK) with a heated electrospray ionization (H-ESI) source. Ionization voltages were set at 3500 V for positive and 3000 V for negative ion modes, respectively. The sheath, auxiliary, and sweep gas flow rates were set to 50, 10, and 1 arbitrary unit (a.u.), respectively. The ion transfer temperature was 320 °C. Resolutions were 70,000 for MS and 17,500 for MS/MS, with a scan range of 100–1500 *m/z*.

#### 2.3.2 GC–MS conditions

The GC–MS conditions for the identification of essential oil compounds were optimized by modifying the protocol described in previous works (Hu et al., 2014; Xu et al., 2020). The GC–MS was performed using an Agilent Technologies 7890B GC System coupled to a 5977B Mass Selective Detector (Agilent Technologies, Santa Clara, CA, USA). An Agilent DB-5MS UI capillary column (30 m × 0.25 mm × 0.25 μm; Agilent J&W Scientific, Folsom, CA, USA) was used for separation. The ionization source operated in electron ionization (EI) mode at 230 °C, with the quadrupole temperature set to 150 °C and an ionization energy of 70 eV. Helium was used as the carrier gas at a constant flow rate of 1.0 mL/min. The injector temperature was maintained at 280 °C, and 1 μL of the sample was injected in split mode (10:1). The oven temperature was programmed as follows: the initial temperature was held at 100 °C for 3 min, followed by a first ramp at 5 °C/min to 150 °C, a second ramp at 1 °C/min to 156 °C with a hold time of 4 min, a third ramp at 2.5 °C/min to 166 °C, and a final ramp at 15 °C/min to 220 °C with a 5-min hold. The post-run was performed at 300 °C for 5 min. The ion source and transfer line temperatures were set to 300 and 280 °C, respectively. The mass spectrometer was operated in full scan mode (50–550 *m/z*).

### 2.4 Untargeted profiling of bioactive compounds in *C. longa* under heat treatment

#### 2.4.1 Peak identification and chemical characterization of the major compounds using UHPLC–HRMS

A Top N data-dependent acquisition (DDA) mode and full scanning mode were employed for MS/MS analysis, which prioritized the most intense precursor ions for fragmentation. Stepped normalized collision energies (NCE) of 10, 30, and 50 were applied during fragmentation to produce a diverse range of product ions, facilitating accurate compound identification and structural characterization. The important untargeted compounds in *C. longa* were identified based on their accurate mass, ion fragmentation pattern, isotopic distribution patterns, and ion fragmentation pattern using Scaffold Elements 2.2.1 (Proteome Software, Portland, OR, USA) and mass spectral libraries. The mass error (ppm) values were calculated using the following equation:

$$\mathrm{Error}\;\left(\mathrm{ppm}\right)=\frac{m\mathrm{easured}\;m/z-\mathrm{expected}\;m/z}{\mathrm{expected}\;m/z}\times 10^{6}$$

To ensure the accuracy of metabolite identification, reference standards were used for the annotation of seven major compounds: bisdemethoxycurcumin, demethoxycurcumin, curcumin, *ar*-turmerone, and 4-vinylguaiacol, vanillin, dehydrozingerone. These standards were analyzed under the same conditions as the samples to confirm their identity based on retention time, MS/MS fragmentation patterns, and accurate mass. For the remaining metabolites, annotation was performed based on high-resolution mass data, MS/MS fragmentation patterns, and compared with online databases such as mzCloud (https://www.mzcloud.org) and MassBank (http://www.massbank.jp/).

For confidence level classification, this study followed the guidelines set by the Metabolomics Standards Initiative (MSI) (Fiehn et al., 2007; Salek, Steinbeck, Viant, Goodacre, & Dunn, 2013). The metabolites were classified into the following categories:

Level 1 (Confirmed identification).

Metabolites confirmed using reference standards, validated by direct comparison of retention time, MS/MS fragmentation patterns, and high-resolution mass accuracy.

Level 2 (Putatively annotated).

Metabolites identified based on ID Score, Isotopic Distribution Score, Mass Accuracy Score, and mass error (ppm), as determined by Scaffold Elements software. These metabolites were further cross-validated through spectral database matching with MassBank and mzCloud, which served as supporting evidence to strengthen identification reliability. However, these metabolites were not directly confirmed using reference standards.

#### 2.4.2 Peak identification and chemical characterization of the essential oils using GC–MS

GC–MS data was processed using the NIST MS Search Program (version 2.3) with the NIST/EPA/NIH Mass Spectral Library (NIST17, National Institute of Standards and Technology, Gaithersburg, MD, USA). Individual compounds were identified by matching their recorded mass spectra to those in the library and by comparing their retention indices (RIs) with values reported in the literature (Avanço et al., 2017). The RIs were determined relative to a C8–C20 alkane standard (Sigma-Aldrich) separated on the HP-5 MS UI capillary column under the same analysis conditions. Each compound was compared using the area ratio, calculated by dividing its peak area by that of the IS.

### 2.5 Statistical analysis

Statistical analysis was performed using MetaboAnalyst 6.0 (http://www.MetaboAnalyst.ca/), a comprehensive metabolomics data analysis platform for multivariate statistical analysis. PCA was done to visualize and compare the metabolic profiles of *C. longa*. The impact of heating duration on the concentration of major compounds was profiled using heatmap cluster analysis to visually represent temporal changes in compound concentrations and the PCA bi-plot approach to reveal the relationships and variations of compounds with heating time. Additionally, one-way ANOVA was used to evaluate the statistical significance of changes in compound concentrations across different heat treatment time points. The results of the ANOVA, including both original and normalized concentration values, were visualized to identify specific compounds that showed significant tendencies to be generated or increased over time during the heat treatment.

### 2.6 Theoretical absorption percentage of the identified major individual compounds

The theoretical absorption percentage and Caco-2 permeability of the identified major individual compounds found in *C. longa*, including degradation compounds during heat treatment, were evaluated as outlined by a previous study (Velderrain-Rodríguez, Quero, Osada, Martín-Belloso, & Rodríguez-Yoldi, 2021). In brief, the theoretical absorption was evaluated through simulations using the chemical structures of the major individual compounds provided in simplified molecular input line entry system (SMILES) codes. The SMILES codes were retrieved from the PubChem Open Chemistry Database (https://pubchem.ncbi.nlm.nih.gov/search/). The molecular properties of the compounds, including molecular weight (MW), topological polar surface area (TPSA), Lipinski's rule of five (LIRF), and theoretical absorption percentage (% Abs), were calculated using Molinspiration (http://www.molinspiration.com/), an online property calculation toolkit. The prediction of the Caco-2 permeability was computed by the pkCSM online platform (http://biosig.unimelb.edu.au/pkcsm/prediction), which employs drug-like molecules with known Caco-2 permeability values to estimate the logarithm of the apparent permeability coefficient (log Papp). The Caco-2 permeability values are expressed as log Papp and reported in units of 10^6^ cm/s.

## 3 Results and discussion

### 3.1 Untargeted profiling of bioactive compounds in *C. longa* under heat treatment

#### 3.1.1 Identification of major compounds using UHPLC–HRMS

Untargeted UHPLC–HRMS analysis of the methanolic extracts of *C. longa* subjected to heat treatment at 180 °C for 0, 10, 30, 60, and 90 min indicated the presence of various key metabolites, including curcuminoids, phenolic compounds, sesquiterpenes, and other bioactive compounds, as summarized in Table 1. During the heat treatment periods, any major metabolite peaks detected at least once, either in Korean ulgeum or Indian turmeric, were identified. The mean peak areas of extracted ions from identified bioactive compounds obtained from three replicates of *C. longa* samples were integrated and compared based on their relative abundances, with detailed results provided in Table S2. For HRMS data processing and metabolite identification, Scaffold Elements software was used, and metabolites were identified based on multiple criteria: ID score, isotopic distribution score, mass accuracy score, mass error (ppm). Only metabolites with an ID score ≥ 0.7 were considered for further analysis. Among these, priority was given to compounds exhibiting high isotopic distribution scores and mass accuracy scores with low mass error (ppm) to ensure reliable metabolite identification.

**Table 1:** Identification of the bioactive compounds in the metabolite profile of dried *C. longa* samples using UHPLC–HRMS.

No.	Identified compounds	ID level^a^	+/−	RT (min)	Molecular Formula	Molecular Weight (g/mol)	Adduct	Expected m/z	Measured *m*/*z*	Error (ppm)	ID score^c^	Mass accuracy score^c^	Isotopic distribution score^c^	Turmeric^d^	Ulgeum^e^	
1	guaiacol	2	+	5.52	C7H8O2	124.1	[M-H]+	125.0603	125.0599	−3.198	0.813	0.82	1	√		
2	vanillic acid	2	−	10.46	C8H8O4	168.2	[M-H]-	167.0344	167.034	−2.604	0.740	0.80	0.92	√	√	
3	vanillin	1	−	14.49	C8H8O3	152.0	[M-H]-	151.0395	151.0388	−4.635	0.876	0.81	0.99	√	√	
4	bisacurone	2	+	16.25	C15H24O3	252.3	[M-H]+	253.1804	253.1802	−0.671	0.838	1	0.97	√		
5	ferulic acid	2	−	18.27	C10H10O4	194.0	[M-H]-	193.0501	193.0498	−1.476	0.740	0.80	0.92	√	√	
6	α-methylcinnamic acid	2	−	20.89	C10H10O2	162.1	[M-H]-	161.0603	161.0596	−4.061	0.959	0.98	1	√	√	
7	dehydrozingerone	1	+	22.14	C11H12O3	192.1	[M-H]+	193.0865	193.0864	−0.363	0.896	0.98	1	√	√	
8	curcumenol	2	+	22.82	C15H22O2	234.2	[M-H]+	235.1698	235.1695	−1.297	0.799	0.99	1	√	√	
9	α-ionone	2	+	24.21	C13H20O	192.2	[M-H]+	193.1592	193.1591	−0.725	0.888	1	0.99		√	
10	*ar*-turmerone	1	+	24.50	C15H20O	216.2	[M-H]+	217.1592	217.1587	−2.487	0.867	1	1	√	√	
11	7-H-1,7-bis(4-H-3-MP)-1-H-3,5-D^b^	2	−	27.99	C21H22O7	386.1	[M-H]-	385.1287	385.1294	1.740	0.795	0.99	0.99	√	√	
12	isocurcumenol	2	+	28.36	C15H22O2	234.2	[M-H]+	235.1698	235.1693	−2.147	0.777	0.86	1	√	√	
13	omega-hydroxymoracin N	2	−	28.57	C19H18O5	326.1	[M-H]-	325.1076	325.1081	1.538	0.899	1	1	√	√	
14	cinnamyl alcohol	2	+	30.93	C9H10O	134.1	[M-H]+	135.0810	135.0807	−2.139	0.790	0.95	0.74	√	√	
15	xanthorrhizol	2	+	31.80	C15H22O	218.2	[M-H]+	219.1749	219.1745	−1.779	0.874	1	1	√	√	
16	3,4-dimethoxycinnamic acid	2	−	32.16	C11H12O4	208.1	[M-H]-	207.0657	207.066	1.280	0.867	0.87	1	√	√	
17	dihydro-bisdemethoxycurcumin	2	+	32.18	C19H18O4	310.1	[M-H]+	311.1283	311.1278	−1.720	0.900	1	1	√	√	
18	guaiazulene	2	+	32.24	C15H18	198.1	[M-H]+	199.1487	199.1484	−1.381	0.864	1	1	√	√	
19	(*S*)-perillic acid	2	+	32.32	C10H14O2	166.1	[M-H]+	167.1072	167.1067	−3.022	0.820	1	0.97		√	
20	bisdemethoxycurcumin	1	+	32.53	C19H16O4	308.1	[M-H]+	309.1127	309.1123	−1.246	0.982	1	1	√	√	
21	dihydro-demethoxycurcumin	2	+	32.59	C20H20O5	340.1	[M-H]+	341.1389	341.1383	−1.759	0.786	0.92	1	√	√	
22	6-prenylnaringenin	2	−	32.63	C20H20O5	340.1	[M-H]-	339.1233	339.1237	1.327	0.955	1	1	√	√	
23	zingerone	2	+	32.66	C11H14O3	194.1	[M-H]+	195.1021	195.1023	0.923	0.780	1	1	√	√	
24	2-methylcinnamic acid	2	−	33.04	C10H10O2	162.1	[M-H]-	161.0603	161.0596	−4.061	0.872	0.76	1	√	√	
25	demethoxycurcumin	1	+	33.05	C20H18O5	338.1	[M-H]+	339.1233	339.123	−0.737	0.981	1	1	√	√	
26	dihydrocurcumin	2	+	33.09	C21H22O6	370.1	[M-H]+	371.1495	371.1494	−0.175	0.900	1	1	√	√	
27	4-vinylguaiacol	1	−	33.57	C9H10O2	150.1	[M-H]-	149.0603	149.0592	−7.071	0.873	0.77	1	√	√	
28	curcumin	1	+	33.58	C21H20O6	368.1	[M-H]+	369.1338	369.1332	−1.666	0.981	1	1	√	√	
29	polygodial	2	+	33.84	C15H22O2	234.2	[M-H]+	235.1698	235.1693	−2.147	0.851	0.98	1	√	√	
30	curcumol	2	+	34.67	C15H24O2	236.2	[M-H]+	237.1855	237.1855	0.190	0.876	0.97	0.96	√	√	
31	2-phenylpropanal	2	+	34.72	C9H10O	134.1	[M-H]+	135.0810	135.0806	−2.880	0.823	0.95	0.99	√	√	
32	costunolide	2	−	34.72	C15H20O2	232.1	[M-H]-	231.1385	231.1386	0.411	0.830	0.99	0.95		√	
33	valerenic acid	2	+	34.96	C15H22O2	234.2	[M-H]+	235.1698	235.1696	−0.850	0.837	0.99	0.99	√	√	
34	farnesal	2	+	38.74	C15H24O	220.2	[M-H]+	221.1905	221.1903	−1.085	0.877	0.97	1	√	√	
35	(+)-trans-chrysanthemic acid	2	+	39.59	C10H16O2	168.1	[M-H]+	169.1229	169.1223	−3.282	0.865	0.99	1	√	√	
36	α-cyperone	2	+	41.30	C15H22O	218.2	[M-H]+	219.1749	219.1744	−2.236	0.956	1	1	√	√	
37	zerumbone	2	+	42.53	C15H22O	218.2	[M-H]+	219.1749	219.1744	−2.236	0.959	1	1	√	√	
38	nootkatone	2	+	42.73	C15H22O	218.2	[M-H]+	219.1749	219.1745	−1.779	0.956	1	1	√	√	

a Confidence level of annotated compounds: 1 (confirmed identification) identified using reference standards; 2 (putatively annotated) identified based on database matches.

b 7-H-1,7-bis(4-H-3-MP)-1-H-3,5-D: 7-hydroxy-1,7-bis(4-hydroxy-3-methoxyphenyl)-1-heptene-3,5-dione.

c ID score, mass accuracy score, and isotopic distribution score were evaluated using Scaffold Elements software. Higher scores indicate greater confidence in metabolite identification.

d Indian-cultivated *C. longa* (turmeric).

e Korean-cultivated *C. longa* (ulgeum).

Among the identified compounds, bisdemethoxycurcumin, demethoxycurcumin, curcumin, *ar*-turmerone, vanillin, dehydrozingerone and 4-vinylguaiacol were classified as MSI level 1 (confirmed identification), while all other annotated compounds were categorized as MSI level 2 (putatively annotated).

The UHPLC–HRMS analysis provided a comprehensive and precise characterization of polar and moderately lipophilic bioactive compounds in *C. longa*, offering critical insights into their stability and transformation under heat treatment. However, due to the complexity of metabolomic data, directly elucidating the correlation between heat treatment and metabolite dynamics based solely on these results remains challenging. Consequently, multivariate statistical analyses were performed to provide a detailed interpretation of these relationships, enabling a deeper understanding of the patterns and interactions between heat-induced changes and metabolite profiles. The results from multivariant statistics analyses are discussed in Section 3.2.

#### 3.1.2 Identification of essential oils using GC–MS

In this study, untargeted profiling was performed using GC–MS under analytical conditions optimized for detecting essential oils. The analysis was conducted in scan mode to enable comprehensive detection of volatile compounds, with a particular focus on sesquiterpenes and other lipophilic constituents. The analysis chromatograms are compiled in Fig. S1. For either Korean ulgeum or Indian turmeric, peaks detected at least once during the heat treatment periods were identified. Among the compounds matching the NIST library with consistent RI values, the ones with the highest probability (%) were selected for identification, as summarized in Table 2. GC–MS data shown in Table 2 was processed using the NIST MS Search Program. Peak identification was performed by matching the obtained mass spectra with the NIST library, selecting compounds with the highest match probability (%), and considering retention index (RI) values to enhance identification accuracy. Moreover, the data processing workflow included peak integration, alignment, and compound identification based on retention times and mass spectra. To ensure accurate comparisons of compound abundances, ethyl octanoate was used as an IS, and the detected peak areas were normalized accordingly. Detailed results of this analysis are provided in Table S3.

**Table 2:** Identification of the essential oil compounds in the metabolite profile of dried *C. longa* samples using GC–MS.

No.	Identified compounds	RT (min)	Molecular Formula	Molecular Weight (g/mol)	Expected m/z	NIST Library#	Retention Index	Turmeric^a^		Ulgeum^b^	
								**Presence**	**Probability (%)**	**Presence**	**Probability (%)**
1	ethyl octanoate as internal standard	6.69	C10H20O2	172	172.1463	63,734	1194	√	95.1	√	92.0
2	α-curcumene	13.74	C15H22	202	202.1722	141,047	1482	√	88.8	√	89.8
3	(−)-zingiberene	14.10	C15H24	204	204.1878	70,231	1496	√	69.6	√	49.7
4	unknown 1	14.36	–	–	–	–	1505	√	–	√	–
5	β-bisabolene	14.46	C15H24	204	204.1878	412,943	1508	√	24.8	√	57.5
6	β-sesquiphellandrene	14.93	C15H24	204	204.1878	122,102	1523	√	65.8	√	54.1
7	β-elemenone	17.32	C15H22O	218	218.1671	249,582	1599	–	29.7	√	33.5
8	unknown 2	17.46	–	–	–	–	1602	√	–	√	–
9	unknown 3	18.66	–	–	–	–	1631	√	–	√	–
10	*ar*-turmerone	19.84	C15H20O	216	216.1514	412,355	1659	√	98.0	√	97.9
11	α-turmerone	20.06	C15H22O	218	218.1671	292,711	1664	√	90.2	√	81.4
12	germacrone	21.14	C15H22O	218	218.1671	413,988	1690	√	9.9	√	79.9
13	β-turmerone	21.58	C15H22O	218	218.1671	408,904	1700	√	68.3	√	72.0
14	bisabolone	23.75	C15H24O	220	220.1827	413,234	1740	√	46.3	√	63.6
15	(*E*)-atlantone	25.41	C15H22O	218	218.1671	57,844	1771	√	86.3	√	36.9
16	curcumenone	28.15	C15H22O2	234	234.1620	414,003	1841	–	–	√	90.5

a Indian-cultivated *C. longa* (turmeric).

b Korean-cultivated *C. longa* (ulgeum).

The relative abundances of most essential oil compounds decreased with prolonged heat treatment in both *C. longa* varieties. The analysis revealed distinct compositional differences in the essential oil profiles, highlighting a key distinction between the *C. longa* varieties. In Indian turmeric, GC–MS analysis showed that *ar*-turmerone had the largest area in terms of relative abundance, while β-turmerone had the second largest area. In contrast, ulgeum exhibited a comparatively broader distribution of major turmerones, including *ar*-turmerone, α-turmerone, and β-turmerone, all of which were present at relatively high abundance. Additionally, Korean ulgeum featured compounds that were absent in Indian turmeric, such as β-elemenone, germacrone, and curcumenone.

The observed differences in essential oil composition between the *Curcuma* species align with findings from previous studies. For instance, research has shown that Korean *C. longa* displayed markedly elevated levels of α-zingiberene (27.7–36.8 %) and β-sesquiphellandrene (13.1–18.2 %) compared to turmeric samples grown in India, Bhutan, and Nigeria (Hwang et al., 2016). This corroborates our observation that Korean ulgeum possesses a broader and richer sesquiterpenoid profile than Indian turmeric. This supports our finding that Korean ulgeum contains a more diverse and abundant range of sesquiterpenoids compared to Indian turmeric.

Unknown compounds (e.g., unknown 1–3) were observed to emerge and increase in abundance during heat treatment. Although these peaks could not be identified using the NIST library, their presence indicates potential degradation or transformation products of existing bioactive compounds during thermal processing.

### 3.2 Exploratory analysis of heat-induced metabolite profiles

#### 3.2.1 Heatmap cluster analysis

**Fig. 1:**
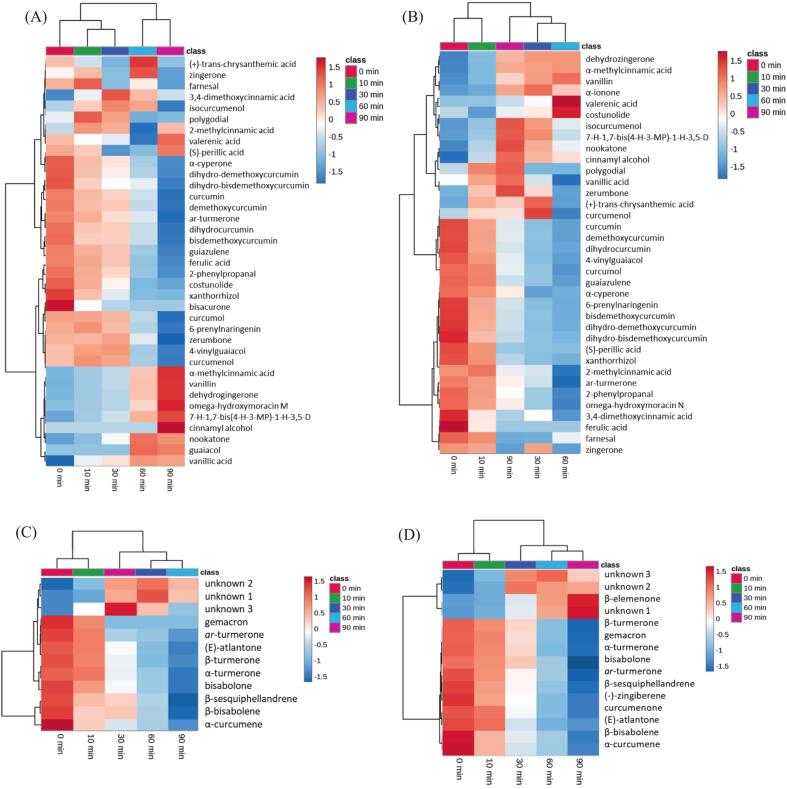
Heatmap cluster analysis results of extracts from *C. longa* varieties subjected to heat treatment at 180 °C for 0, 10, 30, 60, and 90 min: (**A**) UHPLC–HRMS of Indian turmeric methanol extracts, (**B**) UHPLC–HRMS of Korean ulgeum methanol extracts, (**C**) GC–MS of Indian turmeric hexane extracts, and (**D**) GC–MS of Korean ulgeum hexane extracts.

Heatmap cluster analysis (Fig. 1) provides an in-depth visualization of the dynamic changes in metabolite abundance over time and between Korean ulgeum and Indian turmeric, effectively capturing their distinct thermal responses. In *C. longa* varieties, UHPLC–HRMS results revealed a predominance of curcuminoids, including curcumin, demethoxycurcumin, and bisdemethoxycurcumin, which exhibited significant reductions as heat treatment progressed. By 90 min, their levels were drastically diminished, reflecting their high thermal sensitivity and limited stability under prolonged heat exposure. This rapid degradation likely affects their antioxidant potential and bioavailability, which are critical for the functional applications of *C. longa*.

In addition to curcuminoids, *ar*-turmerone, the most abundant sesquiterpene detected in both the UHPLC–HRMS and GC–MS analyses of *C. longa* varieties, demonstrated a steady decline during heat treatment. Unlike the abrupt degradation of curcuminoids, *ar*-turmerone's gradual decrease suggests moderate thermal instability. This degradation is likely associated with a reduction in the aromatic compounds and antimicrobial properties characteristic of *C. longa*.

In contrast to Korean ulgeum and Indian turmeric displayed a more diverse terpenoid profile, with higher initial levels of sesquiterpenes such as *ar*-turmerone, α-turmerone, and β-turmerone. Although these sesquiterpenes also decreased over time, their reduction was less pronounced, indicating relatively greater thermal resilience. Moreover, Korean ulgeum was distinguished by the presence of specific terpenoids, including β-elemenone, curcumenone, and germacrone, which were absent in Indian turmeric. These compounds exhibited distinct thermal transformation patterns; for example, β-elemenone showed a transient increase, peaking between 30 and 60 min of heat treatment, followed by a decline at 90 min. This behavior suggests its formation as an intermediate product of thermal degradation.

These findings highlight the complexity of metabolic transformations in Korean ulgeum compared to Indian turmeric, with Korean ulgeum not only showing the degradation of curcuminoids and turmerones but also the emergence of distinctive bioactive products such as β-elemenone and curcumenone, which were absent in Indian turmeric.

In summary, the heatmap cluster analysis results illustrate the distinct responses of Korean ulgeum and Indian turmeric to thermal processing, emphasizing their unique chemical compositions. While Indian turmeric is predominantly characterized by the rapid degradation of curcuminoids and *ar*-turmerone, Korean ulgeum exhibits greater stability of its terpenoid profile and a broader metabolic diversity under heating. These results provide critical insights into how heat treatment influences the stability and transformation of bioactive compounds in these *C. longa* varieties, offering valuable information for optimizing their functional and nutritional properties in thermally processed applications.

### 3.3 Multivariate statistical analysis of heat-induced metabolite profiles

#### 3.3.1 Principal component analysis (PCA)

**Fig. 2:**
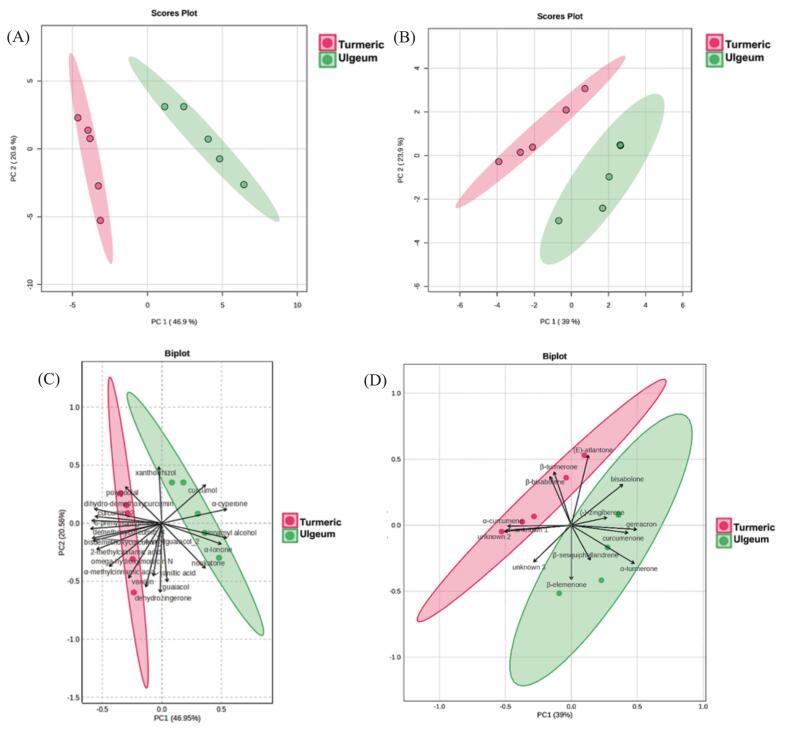
Principal component analysis (PCA) of extracts from two *C. longa* varieties subjected to heat treatment at 180 °C for 0, 10, 30, 60, and 90 min: (**A**) Two-dimensional (2D) plot of methanol extracts analyzed by UHPLC–HRMS, (**B**) 2D plot of hexane extracts analyzed by GC–MS, (**C**) bi-plot of methanol extracts analyzed by UHPLC–HRMS, and (**D**) bi-plot of hexane extracts analyzed by GC–MS.

PCA was performed to interpret the complex datasets obtained from UHPLC–HRMS and GC–MS, aiming to identify the differences in thermal degradation of key metabolites between Korean ulgeum and Indian turmeric and to determine the bioactive compounds contributing to these differences. As shown in Fig. 2(A), the clear separation along PC1 (49.8 %) reflects the distinct metabolic compositions of Korean ulgeum and Indian turmeric. Samples of Indian turmeric clustered separately from those of Korean ulgeum, driven by their relatively higher curcuminoid and phenolic compound levels. Similarly, Fig. 2(B) demonstrates a separation of the two *C. longa* varieties along PC1 (39.0 %) based on their volatile profiles, with PC2 (23.9 %) capturing the temporal shifts caused by heat treatment. The metabolic transformations in Korean ulgeum were more pronounced, likely reflecting its higher abundance of thermally responsive sesquiterpenes and essential oils compared to Indian turmeric.

In Korean ulgeum, the analysis revealed a higher contribution of terpenoids such as *ar*-turmerone, α-turmerone, and β-turmerone to the initial clustering of 0 min samples, as shown in Fig. 2(C). With prolonged heat treatment, decomposition products such as vanillin and dehydrozingerone were observed, suggesting the generation of bioactive degradation products. Furthermore, as shown in Fig. 2(D), terpenoids unique to Korean ulgeum, such as curcumenone and β-elemenone, were more prominent at the later stages of heat treatment, indicating their formation as heat-induced degradation products.

Overall, the distinct metabolic profiles of Korean ulgeum and Indian turmeric, driven by differences in curcuminoid and sesquiterpenoids content. Thermally sensitive compounds such as curcuminoids were more abundant in Indian turmeric, whereas sesquiterpenoids including zingiberene, β-sesquiphellandrene, and α-turmerone were present at higher levels in Korean ulgeum. In addition, heat-induced products specific to each variety were observed, with vanillin, dehydrozingerone, nootkatone, and α-methylcinnamic acid identified in Indian turmeric, and dehydrozingerone, valerenic acid, α-ionone, and β-elemenone detected in Korean ulgeum. Clear temporal shifts in metabolite profiles during heat treatment provide valuable insights into the thermal stability and transformation pathways of bioactive compounds in *C. longa*.

#### 3.3.2 Analysis of variance (ANOVA)

**Fig. 3:**
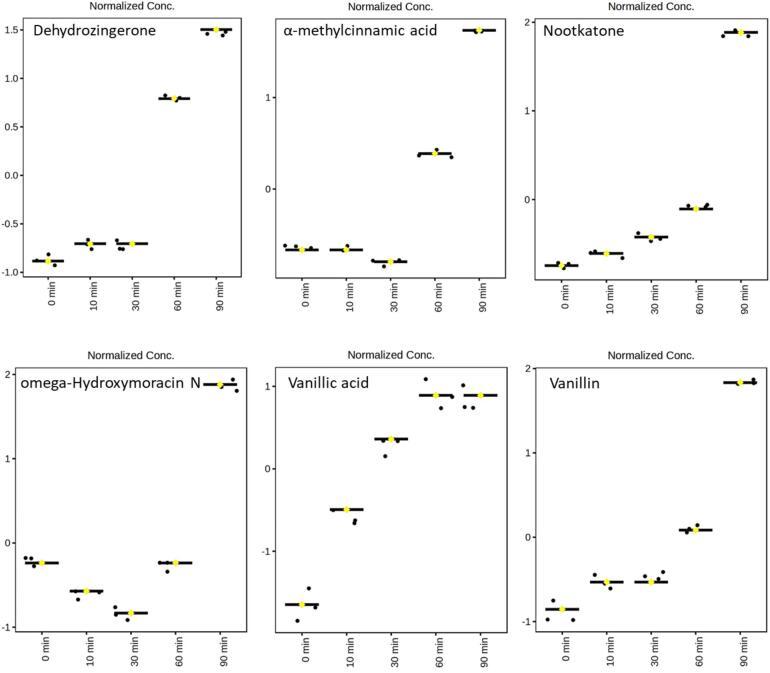
ANOVA results of heat-induced compounds generated or increased during heat treatment at 180 °C (0, 10, 30, 60, and 90 min) in Indian turmeric based on UHPLC–HRMS and GC–MS analyses. Normalized Conc. represents the values after quantile normalization and auto scaling, where quantile normalization adjusts the overall distribution across samples to ensure comparability, and auto scaling standardizes the data by centering the mean at 0 and scaling the standard deviation to 1, enhancing comparability among metabolites. The identified compounds include dehydrozingerone, α-methylcinnamic acid, nootkatone, omega-hydroxymoracin N, vanillic acid, and vanillin.

**Fig. 4:**
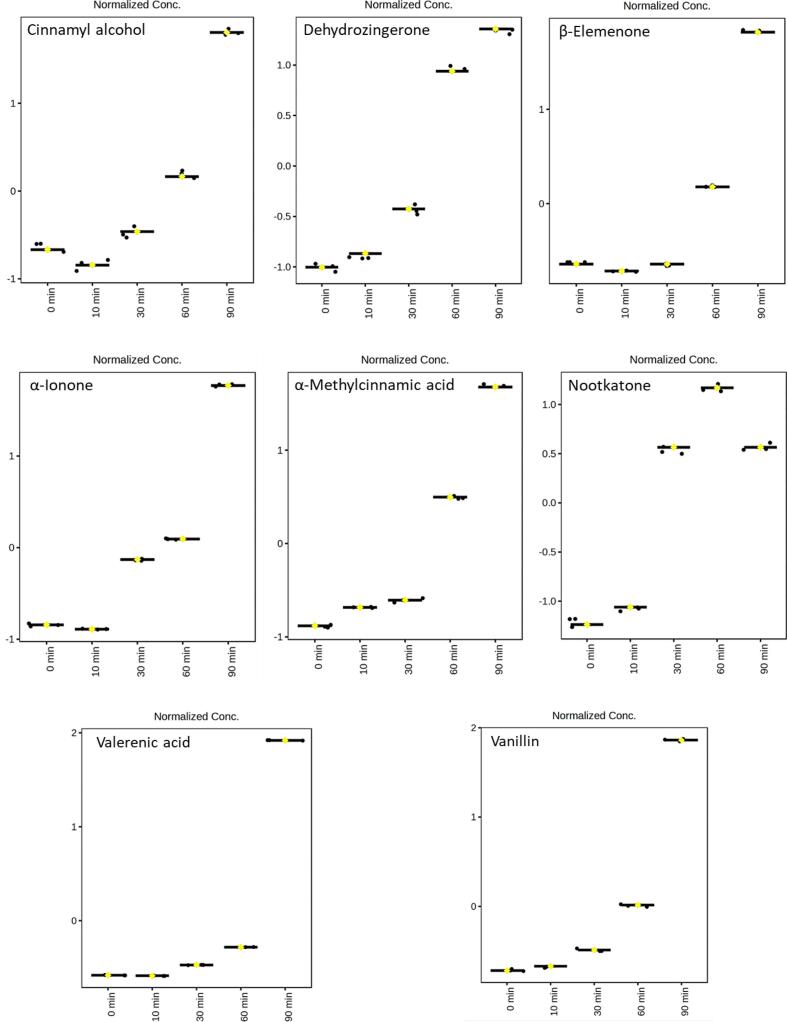
ANOVA results of heat-induced compounds generated or increased during heat treatment at 180 °C (0, 10, 30, 60, and 90 min) in Korean ulgeum based on UHPLC–HRMS and GC–MS analyses. Normalized Conc. represents the values after quantile normalization and auto scaling. The identified compounds include cinnamyl alcohol, dehydrozingerone, β-elemenone, α-ionone, α-methylcinnamic acid, nootkatone, valerenic acid, and vanillin.

*C. longa* is commonly consumed in its heat-processed form, as thermal treatment is a typical step in its preparation as a food product. While major bioactive compounds in *C. longa*, such as curcuminoids, undergo degradation during heat processing, it is possible that these compounds are transformed into other bioactive metabolites rather than losing their biological activity entirely. Therefore, it is essential to characterize and identify the degradation products generated during heat treatment. To address this, ANOVA was performed to identify compounds detected by UHPLC–HRMS and GC–MS that exhibited significant formation or an increasing trend in abundance with heat treatment. The results of this analysis are presented in Fig. 3 and Fig. 4.

The ANOVA analysis identified several compounds with significant changes during heat treatment. In Indian turmeric, dehydrozingerone, nootkatone, α-methylcinnamic acid, ω-hydroxymoracin N, vanillin, and vanillic acid were determined to be statistically significant. Similarly, in Korean ulgeum, significant compounds included nootkatone, dehydrozingerone, valerenic acid, α-ionone, cinnamyl alcohol, α-methylcinnamic acid, vanillin, and β-elemenone. These heat-induced degradation metabolites represent critical candidates for future studies evaluating *C. longa*'s antioxidant, antimicrobial, and anti-inflammatory properties. This study provides foundational data for further exploration of curcuminoids, sesquiterpenes, and their heat-induced degradation products. In particular, vanillin, dehydrozingerone, ferulic acid, and β-elemenone were identified as representative degradation products, contributing to a broader understanding of how thermal processing alters the bioactivity and chemical functionality of *C. longa* varieties.

In previous studies, degradation products of *C. longa* have been identified as ferulic acid, vanillin, dehydrozingerone, 4-vinyl guaiacol, curcumenone, and β-elemenone (Esatbeyoglu et al., 2015; Zhu et al., 2017). In the present study, ferulic acid, 4-vinyl guaiacol, and curcumenone exhibited a slight increase under initial heating conditions, followed by a declining trend after 60 min of heat treatment. According to Slavova-Kazakova et al. (2018), ferulic acid, vanillin, and dehydrozingerone are major degradation products of curcumin. Ferulic acid and vanillin were highlighted as curcumin degradation products with retained antioxidant activity. Although these compounds exhibited lower antioxidant potential than curcumin, they still contributed to *C. longa*'s overall bioactivity (Esatbeyoglu et al., 2015). Dehydrozingerone, formed through the thermal cleavage of curcumin's β-diketone moiety, highlights the generation of bioactive compounds during heat treatment (Hashimoto et al., 2024). Esatbeyoglu et al. (2015) reported that 4-vinyl guaiacol is a significant degradation product of curcumin formed during heat treatment. This compound demonstrated antioxidant and anti-inflammatory properties, indicating that the thermal degradation of curcumin can result in the generation of bioactive metabolites (Esatbeyoglu et al., 2015). These findings align with the thermal degradation patterns observed for *C. longa* varieties in the present study. Furthermore, as mentioned above, previous studies have identified curcumenone and β-elemenone as intermediate or final products formed during heat treatment of *C. longa* (Esatbeyoglu et al., 2015; Zhu et al., 2017). These compounds, which were unique to Korean ulgeum in the present study, highlight the broader transformation pathways and metabolic diversity of this species compared to Indian turmeric (Liu et al., 2023; Zagórska et al., 2023). Their presence reflects the ability of Korean ulgeum to generate novel terpenoid-derived compounds through thermal processes, further emphasizing the distinct chemical responses of the two *C. longa* varieties to heat treatment (Dosoky et al., 2019).

### 3.4 Chemical properties and theoretical absorption percentages of heat-induced metabolites

This study comprehensively evaluated the theoretical absorption percentage and predicted Caco-2 permeability of major bioactive compounds in the two *C. longa* varieties, including essential oils and thermal degradation byproducts, to assess their potential bioavailability and biological activity. As shown in Table 3, curcumin exhibited a relatively low theoretical absorption rate (82.19 %), which can likely be attributed to its high MW and large TPSA, factors that tend to limit cell membrane permeability (Priyadarsini, 2014). This observation aligns with curcumin's low log P_app_ value (−0.093), indicating its relatively restricted Caco-2 permeability compared to its demethylated derivatives (Frank et al., 2017). These findings highlight the critical influence of molecular size and polarity on the bioavailability of curcumin and its derivatives after thermal processing. Curcumin has been reported to have low oral bioavailability due to its rapid metabolism and poor intestinal absorption (Vareed et al., 2008). This finding is consistent with the low theoretical absorption rate predicted in this study.

**Table 3:** Theoretical absorption percentages of identified major bioactive compounds in dried *C. longa* samples based on Lipinski's parameters.

Group	Identified compounds	MW^a^ (g/mol)	TPSA^b^ (Å^2^)	Log P^c^	No. Atoms^d^	Hydrogen Bonds Acceptors	Hydrogen Bonds Donors	Rotatable Bonds	Molecular Volume (Å3)	Violations to LIRF^e^	% ABS^f^	log P_app_^g^ (10^−6^ cm/s)
Curcuminoids	Bisdemethoxycurcumin	308.33	74.6	3.35	23	4	2	6	281.09	0	91.16	0.96
	Demethoxycurcumin	338.35	83.83	3.36	25	5	2	7	306.64	0	91.39	1.02
	Curcumin	368.38	93.07	3.37	27	6	2	8	332.18	0	82.19	−0.09
Flavonoids	omega-Hydroxymoracin N	326.34	94.05	3.70	24	5	4	4	290.66	0	79.31	−0.18
Phenolic compounds	Vanillin	152.15	46.53	1.21	11	3	1	2	136.59	0	84.98	1.22
	Dehydrozingerone	192.21	46.53	2.00	14	3	1	3	180.57	0	93.92	1.24
	α-Methylcinnamic acid	162.19	37.3	2.17	12	2	1	2	155.02	0	100.00	1.73
Phenolic acids	Vanillic acid	168.15	66.76	1.10	12	4	2	2	144.61	0	78.15	0.33
Sesquiterpenes	*ar*-Tumerone	216.32	17.07	4.02	16	1	0	4	230.32	0	94.49	1.46
	Bisacurone	252.35	57.53	2.24	18	3	2	4	258.48	0	94.95	1.26
	Valerenic acid	234.33	37.3	3.79	17	2	1	2	250.16	0	95.52	1.61
	Nootkatone	218.33	17.07	3.90	16	1	0	1	232.13	0	95.64	1.31
	(−)-Zingiberene	204.35	0	4.89	15	0	0	4	234.35	1	95.56	1.42
	β-Bisabolene	204.35	0	5.04	15	0	0	4	234.88	1	95.23	1.42
	β-Sesquiphellandrene	204.35	0	4.89	15	0	0	4	234.9	0	94.67	1.41
	α-Turmerone	218.33	17.07	4.07	16	1	0	4	236.53	0	96.84	1.33
	β-Turmerone	218.33	17.07	4.07	16	1	0	4	237.09	0	96.92	1.29
	Bisabolone	220.35	17.07	4.29	16	1	0	4	242.72	0	96.33	1.26
	(E)-Atlantone	218.33	17.07	4.21	16	1	0	3	236.50	0	96.08	1.34
	Curcumenone	234.33	34.14	3.31	17	2	0	3	239.94	0	96.93	1.58
	Germacron	218.33	17.07	4.36	16	1	0	0	236.48	0	95.52	1.43
	α-Curcumene	202.17	0	4.84	15	0	0	4	228.14	1	93.29	1.54
	β-Elemenone	218.17	17.07	4.07	16	1	0	2	236.84	0	96.70	1.50
Terpenes	α-Lonone	192.30	17.07	3.51	14	1	0	2	208.79	0	95.77	1.51

a MW: Molecular weight. ^b^ TPSA: Topological polar surface area. ^c^ Log P: Octanol–Water partition coefficient (logarithmic scale). ^d^ No. Atoms: Number of atoms. ^e^ Violations to LIRF: Number of violations to Lipinski's rule of five. ^f^ % ABS: Theoretical absorption percentage. ^g^ Log P_app_: Apparent permeability coefficient (logarithmic scale).

Phenolic compounds such as dehydrozingerone and α-methylcinnamic acid demonstrated high absorption percentages (93.92 % and 100 %, respectively) and favorable Caco-2 permeability values (log P_app_: 1.24 and 1.73, respectively). These results suggest that these phenolic compounds are likely to cross the intestinal barrier efficiently, reinforcing their role as major degradation products formed during heat treatment. Similarly, vanillin (84.98 %) and vanillic acid (78.15 %) also displayed favorable absorption percentages, consistent with their known antioxidant properties. The high absorption and permeability of these phenolic acids suggest their potential contribution to *C. longa*'s bioactivity (Ballester et al., 2023).

Among the sesquiterpenes, *ar*-turmerone, β-turmerone, and curcumenone demonstrated high theoretical absorption rates of 94.49 %, 96.92 %, and 96.93 %, respectively, with favorable log P_app_ values of 1.458, 1.292, and 1.584, indicating strong Caco-2 permeability. While studies on the oral or gastric absorption and bioavailability of *ar*-turmerone remain limited, previous researches have reported its anti-inflammatory effects in animal models, and in our study, the in silico prediction also confirmed the high intestinal permeability of *ar*-turmerone, which is consistent with the findings from previous studies (Oh et al., 2014; Park, Jin, Kim, Kim, & Lee, 2012).

Additionally, terpenes such as β-elemenone and α-ionone exhibited exceptional theoretical absorption rates of 96.70 % and 95.77 %, coupled with log P_app_ values of 1.497 and 1.513, respectively, highlighting their superior permeability profiles. These results suggest that these compounds significantly contribute to the bioactive properties of *Curcuma* spp., particularly following heat-induced transformations, underscoring their enhanced bioactivity potential associated with thermal processing.

Overall, these results suggest that the bioactive potential of heat-treated *C. longa* is not solely derived from its original curcuminoids but also from heat-induced degradation products and the essential oils that are more characteristic of Korean ulgeum than Indian turmeric. This study is an initial investigation based on theoretical predictions (in silico modeling), focusing on identifying and selecting degradation byproducts with bioactive potential even after thermal treatment, and suggesting approaches for further investigation. The theoretical absorption modeling has limitations in fully reflecting the complexity of the digestive process, and it is anticipated that the future use of the INFOGEST in vitro digestion model could provide a more realistic evaluation of the behavior of metabolites which undergo thermal treatments in the actual digestive process.

## 4 Conclusion

This study provided data about the chemical transformations of bioactive compounds in the two *C. longa* varieties during heat treatment and characterized newly formed bioactive compounds. In this study, untargeted metabolite profiling was performed using UHPLC–HRMS and GC–MS, enabling a comprehensive analysis of the changes in major metabolites in response to thermal processing. Heatmap cluster analysis and multivariate statistical analysis, including PCA, and ANOVA, highlighted clear metabolic differences between the two *C. longa* varieties. Heat treatment resulted in the degradation of thermally sensitive compounds, such as curcuminoids and turmerones, and different types of heat-induced products were formed between the varieties, including vanillin, dehydrozingerone, nootkatone, α-methylcinnamic acid, valerenic acid, α-ionone, and β-elemenone. These newly generated metabolites are identified as important candidates for further research due to their potential antioxidant, antimicrobial, and anti-inflammatory activities. Phenolic compounds, such as dehydrozingerone and α-methylcinnamic acid, exhibited high theoretical absorption rates (93.92 % and 100 %, respectively) and favorable Caco-2 permeability, supporting their significance as bioactive degradation products formed during heat treatment. Similarly, vanillin and vanillic acid showed strong theoretical absorption properties. Among sesquiterpenes, *ar*-turmerone, β-turmerone, curcumenone, β-elemenone, and α-ionone displayed high theoretical absorption rates and strong intestinal permeability. These findings suggest that heat treatment enhances the bioactive potential of *C. longa* by generating highly bioavailable and biologically active metabolites, contributing to its functionality in food and nutraceutical applications.

## CRediT authorship contribution statement

**Choong-In Yun:** Writing – review & editing, Methodology, Funding acquisition, Conceptualization. **Ga-Yeong Lee:** Investigation, Formal analysis. **Young-Jun Kim:** Writing – review & editing, Project administration, Funding acquisition, Conceptualization. **JaeHwan Lee:** Writing – review & editing, Supervision, Methodology.

## Funding

This research was supported by the 10.13039/501100003725National Research Foundation of Korea (NRF) grant funded by the Korea government (MSIT) (NRF-2022R1F1A1074012), and by the OTOKI Ham TAIHO Foundation.

## Declaration of competing interest

The authors declare that they have no known competing financial interests or personal relationships that could have appeared to influence the work reported in this paper.

## Data Availability

Data will be made available on request.

## Appendix A Supplementary data

Supplementary material Image 1
