# Supplementary material for: Comparative metabolomic profiling using untargeted UHPLC–HRMS and GC–MS reveals thermal-induced chemical changes in dried turmeric
Source: Food Chem X. 2025 Oct 1;31:103104. doi: 10.1016/j.fochx.2025.103104 (PMC12524345; doi:10.1016/j.fochx.2025.103104)
Supplement: Supplementary file 1 — Supplementary material [file mmc1.docx]

Supporting Information for

**Comparative metabolomic profiling using untargeted UHPLC–HRMS and GC–MS reveals thermal-induced chemical changes in dried turmeric**

Choong-In Yun ^a,c^, Ga-Yeong Lee ^b^, Young-Jun Kim ^b,c,*^, JaeHwan Lee ^a,*^

*^a^ Department of Food Science and Biotechnology, Sungkyunkwan University, Suwon 16419, Korea*

*^b^ Department of Food Science and Biotechnology, Seoul National University of Science and Technology, Seoul 01811, Korea*

*^c^ Research Institute of Food and Biotechnology, Seoul National University of Science and Technology, Seoul 01811, Korea*

Corresponding authors.

E-mail addresses: [kimyj@seoultech.ac.kr](mailto:kimyj@seoultech.ac.kr) (Y. Kim), [s3hun@skku.edu](mailto:s3hun@skku.edu) (J. Lee).

| **Page 1** | **Table S1.** Comparison of profiling techniques, statistical analyses, and identified compounds in turmeric samples with previous studies. |
| --- | --- |
| **Page 2 ,3** | **Table S2.** Comparative analysis of the mean peak area from bioactive compounds in three replicates of dried *C. longa* samples at 180°C heating duration, determined by UHPLC–HRMS. |
| **Page 4** | **Table S3.** Comparative analysis of relative levels of identified essential oil compoundsin dried *C. longa* samples at 180°C heating duration determined by GC‒MS. |
| **Page 5, 6, 7** | **Fig. S1.** GC/MS chromatogram of the comparative identified essential oil compounds in dried *C. longa* samples (Indian turmeric and Korean ulgeum) under heating at 180°C. (A), (D), (G), (J), and (M): enlarged views of Indian turmeric, (B), (E), (H), (K), and (N): Indian turmeric, (C), (F), (I), (L), and (O): Korean ulgeum heating durations of 0 min, 10 min, 30 min, 60 min, and 90 min, respectively. Peak assignment: 1 = ethyl octanoate (internal standard); 2 = α-curcumene; 3 = (-)-zingiberene; 4 = Unknown 1; 5 = β-bisabolene; 6 = β-sesquiphellandrene; 7 = β-elemenone; 8 = unknown 2; 9 = unknown 3; 10 = ar-turmerone; 11 = α-turmerone; 12 = germacrone; 13 = β-turmerone; 14 = bisabolone; 15 = (E)-atlantone; 16 = curcumenone. |

**Table S1.** Comparison of profiling techniques, statistical analyses, and identified compounds in turmeric samples with previous studies.

| **No.** | **Scientific name** | **Analytical Instrument** | **Chemometric analysis or Statistical analysis** | **Identified compounds** | **Reference** |  |
| --- | --- | --- | --- | --- | --- | --- |
| 1 | *Curcuma longa* | UHPLC-Q-Orbitrap-HRMS GC‒MS | Heatmap,  PCA^a^,  ANOVA^b^ | Guaiacol, Vanillic Acid, Vanillin, Bisacurone, Ferulic Acid, α-Methylcinnamic Acid, Dehydrozingerone, Curcumenol, α-Ionone, *ar*-Turmerone, Bisdemethoxycurcumin, Dihydrocurcumin, Demethoxycurcumin, 4-Vinylguaiacol, Curcumin, 7-Hydroxy-1,7-Bis(4-Hydroxy-3-Methoxyphenyl)-1-Heptene-3,5-Dione, Isocurcumenol, Omega-Hydroxymoracin N, Cinnamyl Alcohol, Xanthorrhizol, 3,4-Dimethoxycinnamic Acid, Dihydro-Bisdemethoxycurcumin, Guaiazulene, (S)-Perillic Acid, 6-Prenylnaringenin, Zingerone, 2-Methylcinnamic Acid, Polygodial, Curcumol, 2-Phenylpropanal, Costunolide, Valerenic Acid, Farnesal, (+)-Trans-Chrysanthemic Acid, α-Cyperone, Zerumbone, Nootkatone, α-Curcumene, (-)-Zingiberene, β-Bisabolene, β-Sesquiphellandrene, β-Elemenone, α-Turmerone, Germacrone, β-Turmerone, Bisabolone, (E)-Atlantone, Curcumenone | this study |  |
| 2 | *Curcuma zanthorrhiza* | UHPLC-Q-Orbitrap-HRMS | PCA,  PLS-DA^c^ | Leucine, Valine, Phenylalanine, Tryptophan, Zedoarolide B, Zedoalactone B, Curcumenolactone C, Bisacurone, Zedoarol, Zedoaraldehyde, Terpinolene, Camphor, 13-Hydroxygermacrone, Gweicurculactone, Iso-Velleral, *ar*-Turmerone, Coronarin E, Curcumenolactone A, Curzerenone, Curcumene, m-Thymol, Xanthorrhizol, p-Cymene, Gamma bicyclohomo farnesal, Curcumalactone, Agaruspirol, Spathulenol, Elemene, Naringenin, p-Coumaric acid, (+)-Rhododendrol, 4-hydroxybenzoic acid, 2.5-Dimethylphenol, Bisdemethoxycurcumin, Letestuianin A, Demethoxycurcumin, Dihydrocurcumin, Curcumin, Safrole | Klau et al., 2023 |  |
| 3 | *Curcuma longa* L. | UPLC-FT-MS/MS, GC-TOF-MS | PCA,  OPLS-DA^d^,  HCA^e^ | Curcumin, Demethoxycurcumin, Bisdemethoxycurcumin, Dihydrocurcumin, Curcumalongin A, Terpecurcumin A/B/C/E/H/I, Terpecurcumin j, Terpecurcumin D, Terpecurcumin Q/R, Terpecurcumin G/U, Furanodiene, ar-Tumerone, α-Tumerone, Curcumenol, Xanthorrhizol, DAG, PE, PI, PC, PS | Salem et al., 2022 |  |
| 4 | *Curcuma longa, Curcuma zedoaria* | LC-Q-Orbitrap-HRMS | PCA,  PLS-DA | 2,5-Dihydroxybenzoic Acid, 4-Hydroxybenzoic Acid, 4-O-Caffeoylquinic Acid, Caffeic Acid, Carnosol, Chlorogenic Acid, D-(-)-Quinic Acid, Ellagic Acid, Ferulic Acid, Homogentisic Acid, Homoplantaginin, Homovanillic Acid, Nepetin-7-Glucoside, P-Coumaric Acid, Quercetin, Rosmanol, Rutin, Sinapic Acid, Syringic Acid, Trans-Cinnamic Acid, Umbelliferon, Vanillin | Núñez et al., 2020 |  |
| 5 | *Curcuma xanthorrhiza* | QTrap UPLC–MS/MS,  NMR | PCA,  ANOVA,  PLS | Curcumin, Demethoxycurcumin, Bisdemethoxycurcumin, l-Hydroxy-1,7-bis(4-hydroxy-3-methoxyphenyl)-6-heptene-3,5-dione, 1-(4-Hydroxy-3,5-dimethoxyphenyl)-7-(4-hydroxy-3-methoxyphenyl)-1,6-heptadiene-3,5-dione, 1,7-Bis(4-hydroxy-3-methoxyphenyl)-heptane-3,5-diol | Awin et al., 2019 |  |
| 6 | *Curcuma aeruginosa* | LC/MS | PCA,  PLS | *ar*-Turmerone, Curzerene, Furanodiene, Curzerenone, 5-epi-Curzerenone, Furanodienone, Isofuranodienone, Curmadione, Curcumenone, Isocurcumadione, Dehydrocurdione, Germacrone 4,5-epoxide, 13-Hydroxygermacrone, Curcumenol, Epicurcumenol, 4-epi-Curcumenol, Isocurcumenol, Neocurcumenol, Isoprocurcumenol, Neoprocurcumenol, 4-Hydroxy-7(11), 10(14)-guaiadien-8-one, Curcumanolide A, Curcumanolide B, Gajutsulactone A, Gajutsulactone B | Septaningsih et al., 2018 |  |
| 7 | Curcumae Radix | UHPLC/Q-TOFMS | PCA,  OPLS-DA | Curcumin, Curcumenol, Zederone, Neocurdione, Curdione, Curzerenone, Curcumenone, Neocurdione, Curdione | Liu et al., 2016 |  |

^a^ PCA: Principal component analysis. ^b^ ANOVA: One-way analysis of variance, ^c^ PLS-DA: Partial least square discriminant analysis. ^d^ OPLS-DA: Orthogonal projections to latent structures discriminant analysis. ^e^ HCA: Hierarchical cluster analysis.

**Table S2.** Comparative analysis of the mean peak area from bioactive compounds in three replicates of dried *C. longa* samples at 180°C heating duration, determined by UHPLC–HRMS.

| **No.** | **Identified compounds** | **ID level**^a^ | **RT**  **(min)** | **Indian turmeric** | | | | | | **Korean ulgeum** | | | | |  |
| --- | --- | --- | --- | --- | --- | --- | --- | --- | --- | --- | --- | --- | --- | --- | --- |
|  |  |  |  | **0 min** | **10 min** | **30 min** | **60min** | **90min** | **0 min** | | **10 min** | **30 min** | **60min** | **90min** |  |
| 1 | guaiacol | 2 | 5.52 | - | - | - | 823,583 | 764,899 | - | | - | - | - | - |  |
| 2 | vanillic acid | 2 | 10.46 | 508,845 | 796,966 | 863,810 | 989,616 | 968,698 | 459,636 | | 496,100 | 516,868 | 447,479 | 384,874 |  |
| 3 | vanillin | 2 | 14.49 | 1,028,238 | 2,289,285 | 3,183,898 | 6,667,946 | 11,032,603 | - | | 422,406 | 1,270,914 | 1,623,425 | 1,884,618 |  |
| 4 | bisacurone | 2 | 16.25 | 2,421,800 | 649,076 | 193,337 | - | - | - | | - | - | - | - |  |
| 5 | ferulic acid | 2 | 18.27 | 5,040,699 | 4,594,603 | 4,209,734 | 2,624,274 | 1,766,412 | 667,037 | | 250,146 | - | - | - |  |
| 6 | α-methylcinnamic acid | 2 | 20.89 | 10,193,670 | 15,751,904 | 19,111,026 | 33,962,982 | 53,490,783 | - | | 608,508 | 2,118,452 | 2,211,776 | 2,301,243 |  |
| 7 | dehydrozingerone | 2 | 22.14 | 76,568,500 | 107,985,363 | 124,993,649 | 218,301,844 | 328,848,579 | 10,737,068 | | 21,234,443 | 46,985,986 | 51,625,581 | 51,800,971 |  |
| 8 | curcumenol | 2 | 22.82 | 21,322,388 | 25,311,448 | 23,537,768 | 11,690,683 | 3,394,525 | 5,593,544 | | 9,040,963 | 8,836,647 | 14,843,443 | 990,701 |  |
| 9 | α-Ionone | 2 | 24.21 | - | - | - | - | - | 1,986,592 | | 2,976,993 | 13,725,123 | 16,545,702 | 11,842,495 |  |
| 10 | ar-turmerone | 1 | 24.50 | 398,175,298 | 373,692,310 | 348,876,590 | 317,685,120 | 225,204,226 | 283,932,420 | | 266,266,611 | 217,372,312 | 185,074,076 | 90,141,938 |  |
| 11 | 7-H-1,7-bis(4-H-3-MP)-1-H-3,5-D^b^ | 2 | 27.99 | 216,341 | 276,270 | 314,814 | 433,242 | 538,904 | 359,301 | | 544,220 | 1,262,290 | 1,133,045 | 658,186 |  |
| 12 | isocurcumenol | 2 | 28.36 | 8,929,337 | 13,381,398 | 16,606,412 | 15,249,932 | 3,896,477 | 62,549,727 | | 247,751,087 | 989,013,274 | 804,348,601 | 434,432,083 |  |
| 13 | omega-hydroxymoracin N | 2 | 28.57 | 8,003,541 | 8,812,633 | 9,437,858 | 12,790,002 | 18,672,882 | 533,321 | | 481,925 | 405,970 | 308,875 | 213,750 |  |
| 14 | cinnamyl alcohol | 2 | 30.93 | - | - | - | - | 685,131 | 2,019,057 | | 3,368,426 | 5,158,851 | 4,421,648 | 4,062,394 |  |
| 15 | xanthorrhizol | 2 | 31.80 | 441,176,042 | 257,973,419 | 136,688,892 | 22,224,732 | 9,553,185 | 342,778,903 | | 275,543,622 | 79,844,171 | 32,799,957 | 7,718,367 |  |
| 16 | 3,4-dimethoxycinnamic acid | 2 | 32.16 | 43,776 | 58,352 | 75,759 | 66,260 | 55,989 | 43,433 | | 25,342 | 16,485 | 23,846 | 7,697 |  |
| 17 | dihydro-bisdemethoxycurcumin | 2 | 32.18 | 79,412,441 | 67,335,107 | 63,061,741 | 61,224,876 | 45,078,122 | 11,569,029 | | 6,592,423 | 2,621,636 | 920,547 | 314,278 |  |
| 18 | guaiazulene | 2 | 32.24 | 4,924,974 | 4,437,874 | 4,207,591 | 2,954,219 | 1,974,812 | 350,042,623 | | 327,180,839 | 187,922,132 | 143,621,002 | 97,016,335 |  |
| 19 | (S)-perillic acid | 2 | 32.32 | 1,680,433 | 1,433,525 | - | 446,348 | 2,021,267 | 87,247,825 | | 66,983,926 | 8,306,962 | 4,160,865 | 2,607,403 |  |
| 20 | bisdemethoxycurcumin | 1 | 32.53 | 3,348,448,954 | 2,962,097,565 | 2,958,174,371 | 2,640,358,548 | 2,097,164,053 | 215,946,314 | | 147,240,123 | 64,765,197 | 28,214,232 | 8,583,356 |  |
| 21 | dihydro-demethoxycurcumin | 2 | 32.59 | 30,210,728 | 26,245,519 | 24,669,549 | 21,567,818 | 17,595,700 | 14,121,290 | | 9,130,203 | 4,047,426 | 1,551,406 | 450,793 |  |
| 22 | 6-prenylnaringenin | 2 | 32.63 | 71,275,115 | 74,517,746 | 69,136,230 | 55,277,115 | 41,330,943 | 15,067,150 | | 10,293,227 | 4,737,134 | 1,685,961 | 390,857 |  |
| 23 | zingerone | 2 | 32.66 | 677,179 | 786,984 | 449,530 | 997,080 | 357,395 | 618,305 | | 575,194 | - | 588,150 | - |  |
| 24 | 2-methylcinnamic acid | 2 | 33.04 | 1,169,341 | 1,378,931 | 1,343,359 | 969,293 | 1,317,517 | 343,101 | | 360,822 | 245,323 | 236,283 | 114,424 |  |
| 25 | demethoxycurcumin | 1 | 33.05 | 5,450,707,325 | 4,973,091,206 | 4,883,298,110 | 4,231,543,126 | 3,254,758,460 | 428,729,139 | | 327,756,925 | 174,874,683 | 81,686,148 | 24,445,455 |  |
| 26 | dihydrocurcumin | 2 | 33.09 | 446,723,850 | 397,475,223 | 394,643,370 | 348,330,319 | 269,088,638 | 286,320,387 | | 206,469,306 | 101,909,106 | 41,496,924 | 12,187,080 |  |
| 27 | 4-vinylguaiacol | 1 | 33.57 | 62,841,462 | 65,817,820 | 66,552,264 | 52,148,256 | 43,482,884 | 9,502,512 | | 7,795,305 | 3,968,430 | 1,581,410 | 365,233 |  |
| 28 | curcumin | 1 | 33.58 | 10,878,901,992 | 10,135,144,372 | 9,823,093,025 | 8,843,125,741 | 6,960,181,787 | 1,540,382,840 | | 1,149,299,191 | 607,551,586 | 283,642,496 | 95,333,763 |  |
| 29 | polygodial | 2 | 33.84 | 911,333 | 1,603,921 | 1,355,767 | 509,764 | 571,749 | 238,711 | | 756,782 | 853,678 | - | - |  |
| 30 | curcumol | 2 | 34.67 | 1,409,323 | 1,401,431 | 1,370,870 | 1,150,357 | 890,460 | 60,870,709 | | 56,153,349 | 29,174,699 | 16,134,257 | 3,857,795 |  |
| 31 | 2-phenylpropanal | 2 | 34.72 | 583,876,178 | 480,599,426 | 399,222,522 | 180,619,096 | 46,310,450 | 35,549,270 | | 30,896,040 | 21,354,895 | 14,558,203 | 5,680,587 |  |
| 32 | costunolide | 2 | 34.72 | 174,589 | 131,942 | 94,832 | 25,667 | 11,232 | 10,568 | | 8,922 | 11,186 | 11,829 | 15,088 |  |
| 33 | valerenic acid | 2 | 34.96 | 701,307 | 618,074 | 576,689 | 332,120 | 827,851 | 817,579 | | 960,434 | 15,819,969 | 31,545,134 | 97,726,095 |  |
| 34 | farnesal | 2 | 38.74 | 72,244 | 85,004 | 44,593 | 63,169 | 37,775 | 125,824 | | 114,273 | 41,431 | 43,010 | 74,521 |  |
| 35 | (+)-trans-chrysanthemic acid | 2 | 39.59 | 81,094 | 51,831 | 29,299 | 119,131 | 35,648 | 7,951 | | 18,449 | 16,751 | 22,286 | 7,298 |  |
| 36 | α-cyperone | 2 | 41.30 | 1,135,451,764 | 974,376,542 | 913,111,814 | 754,689,064 | 598,081,431 | 1,500,426,433 | | 1,373,680,427 | 1,105,632,960 | 615,011,250 | 670,531,184 |  |
| 37 | zerumbone | 2 | 42.53 | 1,495,036,406 | 1,486,260,392 | 1,516,321,149 | 1,354,200,463 | 1,097,664,140 | 291,150,262 | | 503,457,705 | 652,589,206 | 478,964,014 | 266,605,309 |  |
| 38 | nookatone | 2 | 42.73 | 53,937,880 | 62,009,630 | 79,586,877 | 108,747,094 | 102,935,911 | 19,659,729 | | 42,412,564 | 90,325,566 | 71,406,863 | 51,432,683 |  |

^a^ Confidence level of annotated compounds: 1 (confirmed identification) identified using reference standards; 2 (putatively annotated) identified based on database matches.

^b^ 7-H-1,7-bis(4-H-3-MP)-1-H-3,5-D: 7-hydroxy-1,7-bis(4-hydroxy-3-methoxyphenyl)-1-heptene-3,5-dione

**Table S3.** Comparative analysis of relative levels of identified essential oil compounds in dried *C. longa* samples at 180°C heating duration determined by GC‒MS.

| **No.** | **Identified compounds** | **RT**  **(min)** | **Indian turmeric** | | | | | **Korean ulgeum** | | | | | |
| --- | --- | --- | --- | --- | --- | --- | --- | --- | --- | --- | --- | --- | --- |
|  |  |  | **0 min** | **10 min** | **30 min** | **60min** | **90min** | | **0 min** | **10 min** | **30 min** | **60min** | **90min** |
| 1 | ethyl octanoate as  internal standard | 6.69 | 1.00 | 1.00 | 1.00 | 1.00 | 1.00 | | 1.00 | 1.00 | 1.00 | 1.00 | 1.00 |
| 2 | α-curcumene | 13.74 | 1.69 | 1.31 | 1.15 | 1.06 | 0.86 | | 3.36 | 2.90 | 2.56 | 2.42 | 2.11 |
| 3 | (-)-zingiberene | 14.10 | 0.30 | 0.26 | 0.28 | 0.26 | 0.18 | | 3.43 | 2.91 | 2.46 | 1.88 | 1.48 |
| 4 | unknown 1 | 14.36 | 0.00 | 0.04 | 0.20 | 0.27 | 0.18 | | 0.00 | 0.03 | 0.10 | 0.19 | 0.28 |
| 5 | β-bisabolene | 14.46 | 0.21 | 0.17 | 0.17 | 0.13 | 0.09 | | 0.80 | 0.68 | 0.60 | 0.55 | 0.46 |
| 6 | β-sesquiphellandrene | 14.93 | 0.73 | 0.60 | 0.57 | 0.45 | 0.27 | | 4.19 | 3.66 | 3.12 | 2.48 | 1.81 |
| 7 | β-elemenone | 17.32 | 0.00 | 0.00 | 0.00 | 0.00 | 0.00 | | 0.37 | 0.46 | 0.99 | 1.66 | 2.20 |
| 8 | unknown 2 | 17.46 | 0.14 | 0.51 | 1.26 | 1.41 | 1.15 | | 0.23 | 0.54 | 1.74 | 1.59 | 1.50 |
| 9 | unknown 3 | 18.66 | 0.00 | 0.10 | 0.22 | 0.14 | 0.06 | | 0.00 | 0.26 | 0.81 | 0.89 | 0.64 |
| 10 | ar-turmerone | 19.84 | 22.63 | 20.87 | 14.58 | 13.98 | 11.87 | | 19.66 | 18.54 | 16.58 | 14.81 | 12.24 |
| 11 | α-turmerone | 20.06 | 1.86 | 1.78 | 1.18 | 0.92 | 0.51 | | 17.54 | 16.10 | 12.97 | 7.49 | 4.13 |
| 12 | gemacron | 21.14 | 0.21 | 0.16 | 0.00 | 0.00 | 0.00 | | 4.99 | 4.57 | 3.31 | 1.60 | 0.27 |
| 13 | β-turmerone | 21.58 | 6.42 | 5.64 | 3.25 | 1.67 | 0.63 | | 8.73 | 8.00 | 6.46 | 3.95 | 1.95 |
| 14 | bisabolone | 23.75 | 0.22 | 0.18 | 0.13 | 0.10 | 0.05 | | 1.77 | 1.69 | 1.48 | 1.09 | 0.64 |
| 15 | (E)-atlantone | 25.41 | 0.75 | 0.68 | 0.42 | 0.23 | 0.08 | | 3.86 | 3.52 | 1.58 | 0.66 | 0.28 |
| 16 | curcumenone | 28.15 | 0.00 | 0.00 | 0.00 | 0.00 | 0.00 | | 5.10 | 4.75 | 3.13 | 2.05 | 1.19 |

All values represent the relative abundance of each compound, calculated as the ratio of compound peak area to that of the internal standard (ethyl octanoate).

Data are expressed as the mean of three replicates.


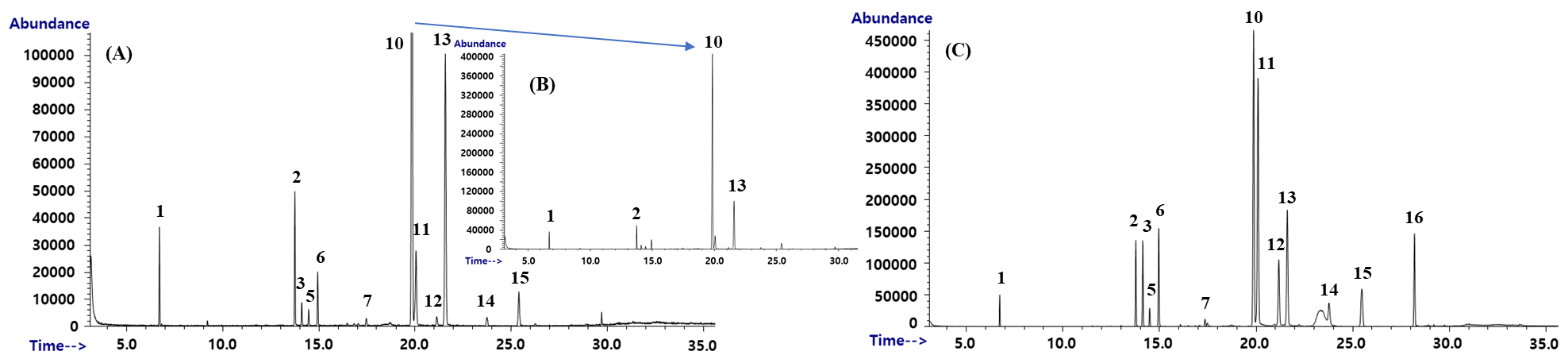


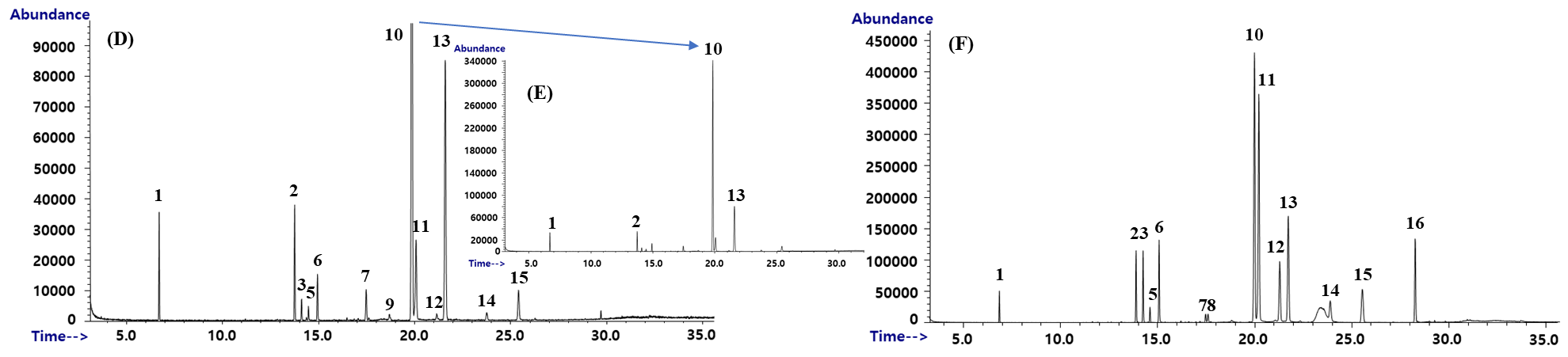


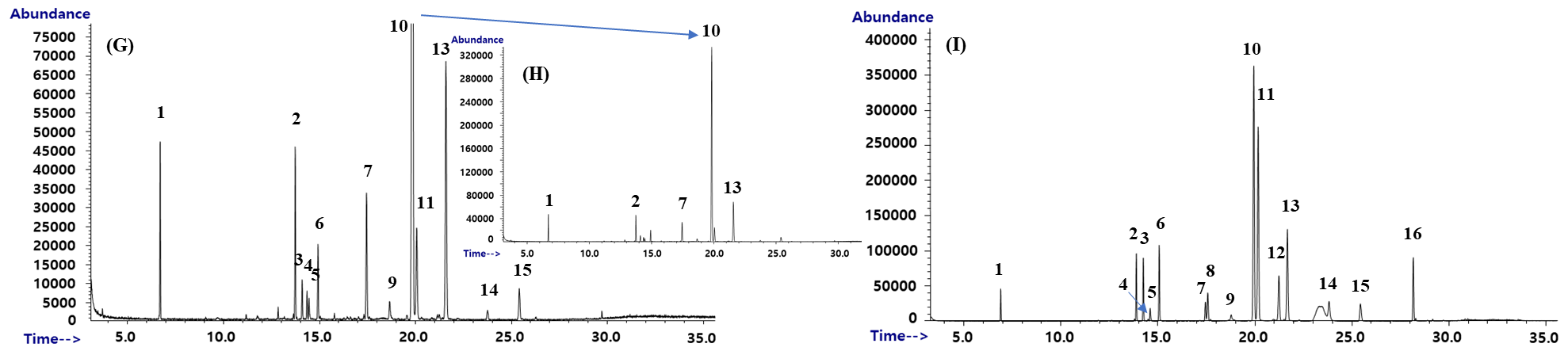


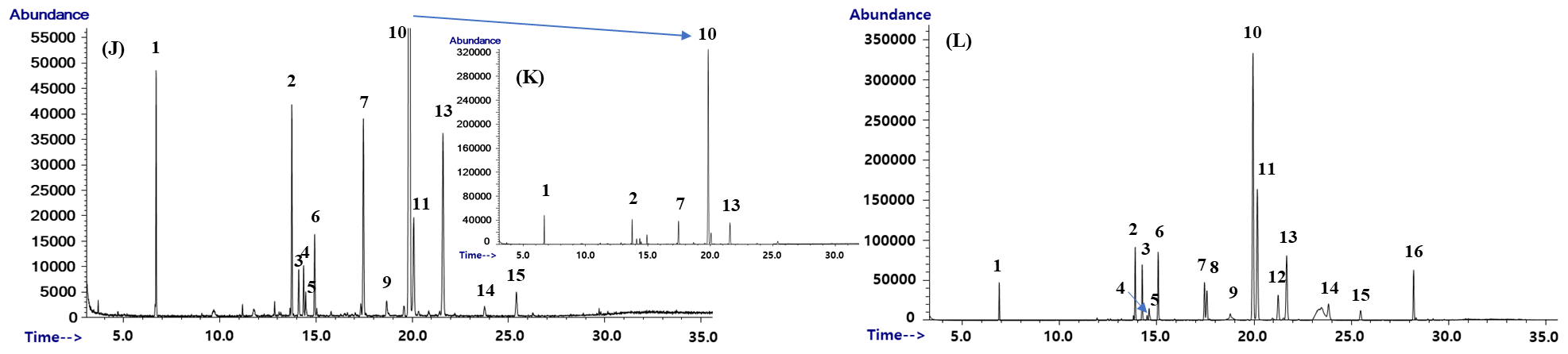


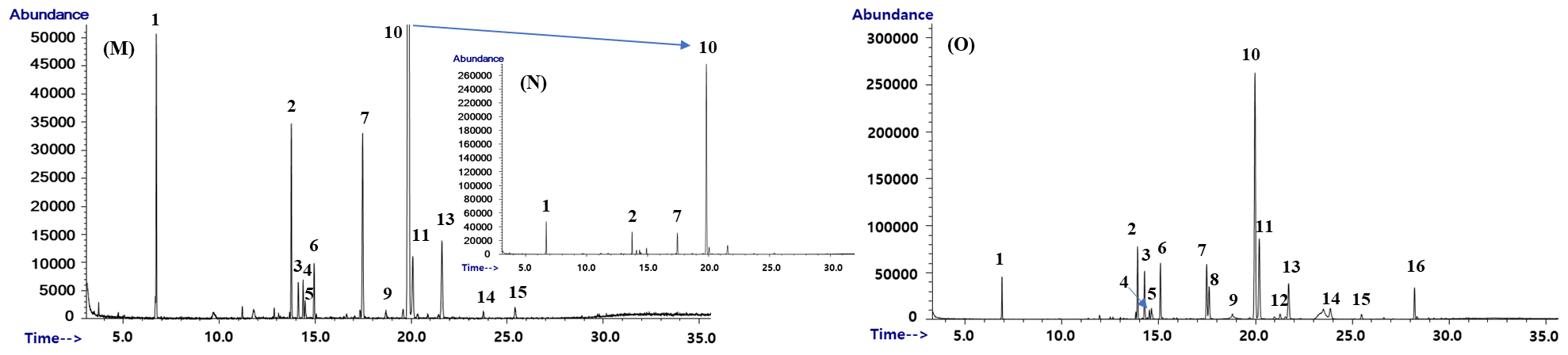


**Fig. S1.** GC/MS chromatogram of the comparative identified essential oil compounds in dried *C. longa* samples (Indian turmeric and Korean ulgeum) under heating at 180°C. **(A), (D), (G), (J), and (M):** enlarged views of Indian turmeric, **(B), (E), (H), (K), and (N):** Indian turmeric, **(C), (F), (I), (L), and (O):** Korean ulgeum heating durations of 0 min, 10 min, 30 min, 60 min, and 90 min, respectively. **Peak assignment:** 1 = ethyl octanoate (internal standard); 2 = α-curcumene; 3 = (-)-zingiberene; 4 = Unknown 1; 5 = β-bisabolene; 6 = β-sesquiphellandrene; 7 = β-elemenone; 8 = unknown 2; 9 = unknown 3; 10 = *ar*-turmerone; 11 = α-turmerone; 12 = germacrone; 13 = β-turmerone; 14 = bisabolone; 15 = (E)-atlantone; 16 = curcumenone.
